# The risk of iodine deficiency in the current model of iodine prophylaxis in Poland

**DOI:** 10.1186/1756-6614-6-S2-A61

**Published:** 2013-04-05

**Authors:** Zbigniew Szybiński

**Affiliations:** 1 The Polish Council for Control of Iodine Deficiency Disorders

## 

Poland is an iodine deficient area. Polish Council for Control of Iodine Deficiency Disorders (PCCIDD) as a multicenter group of experts in the field, was set up in 1991 at the Department of Endocrinology, Jagiellonian University, Collegium Medicum in Kraków. PCCIDD organized in 1992/1993 epidemiologic survey which confirmed iodine deficiency and endemic goiter on the population level and applied to the Ministry of Health for introducing an obligatory model of iodine prophylaxis based on the household salt iodization. In 1996 the Ministry of Health issued such disposition with 20-40 mg KJ per 1 kg of household salt. The council detailed also other elements of iodine prophylaxis, including to the model an iodization of newborn formula (0.10-0.15 mg KJ/l) for children not being breastfed, and formulated recommendation for additional iodine supplementation in pregnant and breast-feeding women with 100-150 µg of KI/day in tablets. In 5-10 years after implementation of this model, the prevalence of goiter in children aged 6-12 years fell from 24.5% to 4.7% - below endemic levels, in pregnant women from 80% to 19%, and frequency of TSH, over 20 µUI/ml in blood of neonates fell from 2.0% to 0.14%. Sufficient dose of iodine on the population level, became an important protective factor in the case of nuclear accident. However, the Technical Consultation WHO in Paris (2006) and in Luxembourg (2007) introduced the need to restrict daily salt consumption, being a risk factor for hypertension and atherosclerosis, to 5.0 grams per person. Hypertension currently affects in Poland approximately nine million persons. Complications associated with hypertension are currently the leading causes of death among those aged 60 years and over. Actual salt consumption in Poland is highest in Europe (15.0 g/day/person) and should be reduced - at least - by 50%. It may create a real risk of iodine deficiency on the population level again, and it is necessary to introduce additional standardized carriers of iodine. Actually we have two such products available on the market: iodized mineral water “Ustronianka z Jodem” (150 µg of iodine/l) and “Wysowianka” (200 µg/ iodine/l), and cow's milk (140-160 µg/ iodine/l) – especially important for schoolchildren and pregnant and breast-feeding women. The control of effectiveness of this model is developed within the National Program for Elimination of Iodine Deficiency financed by the Ministry of Health. This program is multicenter (Warszawa, Łódź, Katowice) and coordination of this multicenter action is performed in the Department of Endocrinology UJCM in Kraków.

